# A Broadband Microwave Absorber Operating Under High-Power Antenna Irradiation

**DOI:** 10.3390/mi17101113

**Published:** 2026-09-24

**Authors:** Wenjun Yu, Dong Yang, Zhong Cheng, Zheyu Zhao, Junyi Nangong, Tiancheng Han

**Affiliations:** 1 Chengdu Aircraft Industrial (Group) Co., Ltd., Chengdu 610073, China; 2 National Center (Sichuan) of Technology Innovation for Advanced Aviation Equipment Corporation, Chengdu 610073, China; chengz004@avic.com; 3 Key Laboratory of Multi-Spectral Absorbing Materials and Structures of Ministry of Education, National Engineering Research Center of Electromagnetic Radiation Control Materials, University of Electronic Science and Technology of China, Chengdu 611731, China; zhaozheyu1102@163.com (Z.Z.); 202611020212@std.uestc.edu.cn (J.N.)

**Keywords:** broadband microwave absorption, electromagnetic–thermal coupling, periodic resistive film, thermal management, frequency selective surface

## Abstract

A microwave absorber is important for reducing the scattering of airborne antennas and radio-frequency systems. However, absorbed electromagnetic energy is inevitably converted into heat, and high-power antenna irradiation may cause local temperature rise and safety hazards. In this work, we demonstrated how to overcome this bottleneck to be compatible with broadband absorption and to work under high-power electromagnetic irradiation. Through the analysis of the electromagnetic–thermal characteristics of typical periodic structural units, cross-shaped and square-ring resistive-film patterns were chosen to design a broadband absorber. Finally, a dual-layer structure consisting of a lower square-ring resistive-film layer and an upper cross-shaped resistive-film layer was designed using equivalent-circuit modeling and full-wave simulation. The fabricated absorber exhibited a measured reflection response below −10 dB from 5.1 to 18 GHz. When the absorber is illuminated by a high-power antenna, its steady-state temperature is only 59.7 °C. These results indicate that the proposed scheme can balance broadband microwave absorption and thermal-management capability, providing a feasible route for absorbers used in high-power antenna irradiation environments.

## 1. Introduction

Radar absorbing structures are widely used to reduce the electromagnetic scattering of airborne platforms [1]. For airborne antennas and radio-frequency systems, absorbing structures can be introduced to suppress undesired reflections and reduce the radar scattering signature [2,3]. Lightweight honeycomb and sandwich absorbers are attractive for airborne applications because they can combine electromagnetic attenuation with structural support [4,5]. Patterned aramid-paper honeycomb structures have also been developed to integrate frequency-selective responses into lightweight structural carriers [6]. Aramid-paper-based absorbers have further shown the potential for lightweight wideband electromagnetic absorption [7]. However, electromagnetic absorption is intrinsically accompanied by energy conversion into heat, and high-power electromagnetic irradiation can lead to significant temperature rise in absorbing materials and structures [8,9]. As illustrated in Figure 1, the absorber considered in this work is therefore required to provide broadband absorption while suppressing electromagnetic energy deposition and maintaining thermal-management capability in the antenna irradiation environment.

Periodic resistive-film absorbers and frequency selective surfaces (FSSs) provide a flexible route for designing thin and tunable microwave absorbers. Their electromagnetic response can be adjusted by changing the pattern topology, geometrical dimensions, and sheet resistance. Related frequency-selective absorbers have shown that multilayer FSS-type configurations can provide compact platforms for tailoring frequency-dependent electromagnetic responses [10]. Resistive-film-based FSS absorbers with patterned conductive films have demonstrated broadband microwave absorption performance [11]. Conductive thin-film layers, such as ITO-based surfaces, have also been used to construct ultra-thin broadband absorbers, indicating that functional-film parameters can be used to tune microwave absorption responses [12]. Double-layer resistor-loaded FSS absorbers and printed lossy capacitive surfaces have also been used to realize broadband microwave absorption [13,14]. Complex surface-impedance absorbers and FSS-honeycomb composites further show the potential of surface-impedance regulation in lightweight broadband absorber design [15,16]. Equivalent-circuit models are also useful for linking FSS geometries with resonance frequencies, surface impedances, and absorption responses [17,18].

Under high-power electromagnetic irradiation, the absorbed energy may cause temperature rise and heat accumulation [19,20]. Multiphysics analyses have been performed to evaluate the thermal response and heat-accumulation behavior of high-power microwave absorbers [21]. It is indicated that local electric-field enhancement and electromagnetic–thermal coupling can affect the thermal robustness of frequency-selective structures [22,23]. Recently, high-temperature-resistant substrates are utilized for electromagnetic response control, further indicating the importance of thermal management [24].

In this work, a dual-layer periodic resistive-film absorber is designed as a broadband microwave absorber operating under high-power antenna irradiation. The proposed absorber aims to balance broadband absorption and thermal-management capability. Through the analysis of the electromagnetic–thermal characteristics of typical periodic structural units, cross-shaped and square-ring resistive-film patterns were chosen to design a broadband absorber. The fabricated absorber exhibited a measured reflection response below −10 dB from 5.1 to 18 GHz. When the absorber is illuminated by a high-power antenna, its steady-state temperature is only 59.7 °C. The measured results agree well with the simulation results, which verifies the proposed design.

## 2. Thermal Response of Periodic Resistive-Film Patterns

To clarify the influence of periodic resistive-film patterns on local thermal risk, electromagnetic–thermal coupled analysis was first carried out for typical patterns. Figure 2a,b show the cross and square-ring patterns, together with their main geometrical parameters. Here, *l* denotes the characteristic length of the pattern, and *w* denotes the strip width or ring width.

The two patterns share the same single-layer absorber unit model. The bottom layer is a dielectric layer with a thickness of *h* = 4.6 mm, and its relative permittivity is ${\varepsilon_{r}}^{\prime }$ = 1.1. A 50 μm-thick PI film is placed on the dielectric layer as the printing substrate, with ${\varepsilon_{r}}^{\prime }$ = 3.9. The top layer is a periodic resistive-film pattern with a specified sheet resistance *R_s_*. By varying the sheet resistance, characteristic length, and strip or ring width, the effects of key structural parameters on the maximum temperature can be analyzed. Specifically, the reference parameters of the cross pattern were *l* = 9.8 mm, *w* = 0.6 mm, and *R_s_* = 200 Ω/□. The reference parameters of the square-ring pattern were *w* = 1 mm, *l* = 9.8 mm, and *R_s_* = 100 Ω/□. The period of both patterns was fixed at *p* = 10 mm.

To ensure the comparability of different parameter effects, the electromagnetic–thermal coupled analysis was performed under the same irradiation and heat-dissipation conditions. The incident frequency was fixed at 1 GHz, and the power density was 20 kW/m^2^. The thermal conductivity of the dielectric layer was set to *κ* = 0.02 W/(m·K). Periodic boundary conditions were applied to the unit cell. Thermal radiation and convection were considered on the upper and lower surfaces. The infrared emissivity was set to *ε*_IR_ = 0.9, and the convective heat transfer coefficient was set to *h_c_* = 10 W/(m^2^·K). Under these conditions, the maximum temperature was simulated.

It is known that electromagnetic energy is mainly converted into heat through Ohmic loss in the resistive film. The average loss power density can be written as [23].

(1) $$P_{jav}=\frac{1}{2}\sigma |E{|}^{2}$$

where *σ* is the equivalent conductivity of the resistive film, and *E* is the local electric field intensity. Therefore, the heat generation is determined not only by the electrical conductivity of the material, but also by the local electric field intensity.

Figure 2c–e show the effects of sheet resistance, characteristic length *l*, and strip/ring width *w* on the maximum temperature of the cross and square-ring patterns. These parameters simultaneously influence the electromagnetic loss, impedance matching, current distribution, and equivalent circuit characteristics of the resistive films, resulting in different thermal responses. The maximum temperature exhibits parameter-dependent variations, including both monotonic and non-monotonic trends. Specifically, increasing sheet resistance changes the balance between Ohmic loss and impedance matching, while increasing the characteristic length strengthens electromagnetic coupling by enlarging the pattern occupation ratio and edge-field interaction. Meanwhile, the strip/ring width modifies the resistive-film area and current path, leading to variations in the surface current distribution and thermal response. Therefore, the electrical and geometrical parameters of resistive-film patterns should be optimized by considering both broadband absorption performance and temperature control rather than maximizing electromagnetic loss alone.

## 3. Design of the Dual-Layer Broadband Absorber

Single-layer periodic resistive-film absorbers usually suffer from limited absorption bandwidth, making it difficult to simultaneously achieve broadband absorption and thermal-management requirements [13,25]. To overcome this limitation, a dual-layer periodic resistive-film absorber was designed for broadband absorption and thermal management under antenna irradiation in this work. The cross-shaped and square-ring FSS layers provide frequency-dependent electromagnetic responses, enabling broadband absorption while reducing electromagnetic energy deposition.

A periodic resistive-film pattern can be regarded as a resistive FSS with frequency-selective characteristics [17,18]. The FSS layer is modeled as a surface impedance element, while the dielectric layers are modeled as transmission-line sections. Together, they determine the input impedance and reflection response of the absorber. When the FSS pattern shows a clear resonant behavior, its surface impedance can be approximated by a series RLC circuit [17]:

(2) $$Z_{FSS}(\omega )=R+j\omega L+\frac{1}{j\omega C}$$

where *R* represents the Ohmic loss of the resistive film, while *L* and *C* are the equivalent inductance and capacitance of the pattern, respectively. Compared with direct full-wave parameter sweeping, the equivalent-circuit model links the pattern dimensions, sheet resistance, dielectric thickness, resonance frequency, and absorption bandwidth. Both the square-ring and cross patterns used in this work exhibit typical resonant FSS characteristics, and they can be modeled by series RLC branches.

For the square-ring FSS, the surface current along the ring arms contributes to magnetic energy storage. The electric field concentrated in the gaps contributes to capacitive energy storage. The resistive film introduces Ohmic loss, and the equivalent parameters of the square-ring FSS can be written as [18]:

(3) $$R_{ring}=R_{s}\frac{p^{2}}{{\left(p-g\right)}^{2}}\frac{p-g}{2w}exp\left[A\left(1-\frac{g+2w}{p}\right)\right]$$

(4) $$L_{ring}=\frac{1}{\omega }\frac{p-g}{p}F(p,2w,\lambda )$$

(5) $$C_{ring}=\frac{4}{\omega }\frac{p-g}{p}F(p,g,\lambda )$$

where *R_s_* is the sheet resistance of the square-ring resistive film, *p* is the period, *w* is the ring width, *g* is the gap between adjacent square rings, *A* is an empirical fitting constant, and *F*(*p*,*w*,*λ*) is a correction function related to the period, ring width, and wavelength. These expressions indicate that the equivalent *R*, *L*, and *C* of the square-ring layer are jointly determined by the sheet resistance, period, ring width, and gap.

For the cross-shaped FSS, under normal incidence, the arm parallel to the incident electric field mainly contributes to conduction and energy storage. The equivalent resistance is determined by the arm length and width. The equivalent inductance is mainly related to the current path, while the equivalent capacitance is associated with the gap coupling between adjacent arms. The equivalent parameters of the cross-shaped FSS can be written as [26].

(6) $$R_{cross}=R_{s}\frac{p^{2}}{lw}$$

(7) $$L_{cross}=\frac{1}{\omega }H(p,w,\lambda )=\frac{1}{\omega }\frac{p}{\lambda }\cos\theta \left[\ln\left(\csc\frac{\pi w}{2p}\right)+G(p,w,\lambda )\right]$$

(8) $$C_{cross}=\frac{4}{\omega }\left[\frac{w}{p}H(p,g,\lambda )+\frac{l}{p}H(p,p-w,\lambda )\right]$$

where *R_s_* is the sheet resistance of the cross-shaped resistive film, *l* is the arm length, *w* is the arm width, *p* is the period, and *G*(*p*,*w*,*λ*) is a correction factor. These relations indicate that the equivalent circuit parameters are determined by the electrical and geometrical parameters of the cross pattern.

Based on the above equivalent relations, the dielectric-layer thicknesses and the equivalent *R*, *L*, and *C* parameters of the two resistive-film layers were first optimized in the circuit model. The optimized circuit parameters were then mapped to the actual geometrical dimensions of the square-ring and cross patterns. Full-wave simulation was then used to verify the reflection response of the designed absorber. For a multilayer absorber backed by a metal ground, the reflection response in decibels can be expressed as [27].

(9) $$R_{dB}=20\log|\Gamma |=20\log\left|\frac{Z_{in}-Z_{0}}{Z_{in}+Z_{0}}\right|$$

where *Z*_in_ is the input impedance of the absorber, and *Z*_0_ is the characteristic impedance of free space. Therefore, the design of the dual-layer periodic resistive-film absorber is essentially to tune the equivalent *R*, *L*, and *C* parameters of the two patterned layers, together with the dielectric-layer thicknesses. The objective is to approach impedance matching in the target high-frequency band while reducing low-frequency electromagnetic energy deposition and heat accumulation.

Figure 3 shows the physical structure and equivalent-circuit model of the designed dual-layer periodic resistive-film absorber. The absorber is backed by a metal ground and consists of a lower square-ring resistive-film layer and an upper cross-shaped resistive-film layer separated by low-permittivity dielectric layers (${\varepsilon_{r}}^{\prime }$ = 1.1). The resistive films are printed on 50 μm PI film substrates (${\varepsilon_{r}}^{\prime }$ = 3.9). The optimized structure has a period of *p* = 9 mm, with dielectric thicknesses of *h*_1_ = 3.6 mm and *h*_2_ = 4.9 mm. Other geometrical parameters of the resistive-film patterns can be obtained from the unit-cell schematic in Figure 3a. The upper cross layer improves impedance matching and provides high-frequency absorption, while the lower square-ring layer contributes to the low- and middle-frequency response.

The optimized equivalent parameters of the square-ring layer are *R* = 107.5 Ω, *L*_0_ = 0.3 nH, and *C*_0_ = 0.195 pF, while those of the cross-shaped layer are *R* = 397 Ω, *L*_0_ = 8.75 nH, and *C*_0_ = 0.015 pF. Figure 3c compares the equivalent-circuit prediction with the full-wave simulation, showing good agreement in the main absorption band. The full-wave simulation shows that the reflection response remains below −10 dB from 5 to 18 GHz, indicating good high-frequency broadband absorption. Figure 3d compares the reflection responses of the individual and combined layers. The square-ring and cross layers provide complementary frequency responses, and their combination extends the absorption bandwidth through multilayer electromagnetic coupling.

## 4. Loss Distribution and Low-Frequency Thermal Response

To further clarify the absorption mechanism of the dual-layer structure, the loss power density distributions in the square-ring and cross layers were extracted at representative frequencies, as shown in Figure 4. The selected frequencies correspond to the main absorption regions in Figure 3c, representing low-, intermediate-, and high-frequency responses.

At 6 GHz, the loss is mainly concentrated in the lower square-ring layer, indicating that the low-frequency response is dominated by the square-ring layer. As the frequency increases, both layers contribute to the absorption around 11 GHz, while the cross layer becomes dominant at 16 GHz. These results demonstrate that the dual-layer absorber achieves frequency-dependent loss distribution, where different resistive-film layers contribute to different frequency ranges.

To evaluate the thermal response of the dual-layer structure under high-power irradiation, steady-state thermal simulations were carried out at several typical frequency points. The power density was set to 20 kW/m^2^. At 0.8, 1.0, 1.3, and 1.8 GHz, the maximum temperatures of the lower square-ring layer were 48.4, 61.7, 83.5, and 116 °C, respectively, while those of the upper cross layer were 38.5, 47.7, 62.9, and 85 °C. To further evaluate the thermal-management capability of the proposed absorber, a reference single-layer resistive-film absorber was constructed based on the crossed-ring resistive-film absorber reported in [11]. The unit-cell configuration and geometric parameters of the reference absorber are shown in Figure 5a, where the sheet resistance of the resistive film was set to 75 Ω/□. To ensure a fair thermal comparison, a PI substrate with the same thickness as that of the proposed absorber was introduced into the reference structure. The electromagnetic and thermal parameters of the PI substrate were set identical to those of the proposed absorber. Similarly, the PMI dielectric layer was assigned the same electromagnetic and thermal properties as those used in the proposed absorber.

The simulated reflection response of the proposed absorber and the reference absorber is shown in Figure 5b. The reference absorber exhibits a −10 dB absorption bandwidth from approximately 6 to 16 GHz, indicating that it maintains effective broadband microwave absorption capability and can serve as a representative high-frequency absorbing structure. Under the same irradiation condition at 1 GHz, electromagnetic–thermal simulations were further performed for both absorbers. As shown in Figure 5c,d, the maximum temperature of the reference absorber reached 307 °C, which was significantly higher than that of the proposed absorber (61.7 °C). These results demonstrate that, compared with conventional resistive-film absorbers without specific thermal–management design, the proposed dual-layer structure effectively suppresses temperature rise under low-frequency high-power irradiation conditions.

## 5. Fabrication and Experimental Verification

To verify the manufacturability, electromagnetic performance, and thermal-management capability of the designed dual-layer periodic resistive-film absorber, sample fabrication, reflection measurement, and low-frequency high-power irradiation tests were carried out. A dual-layer periodic resistive-film absorber with a size of 300 mm × 300 mm was fabricated by screen printing patterned resistive films on thin PI substrates. The dielectric layer was composed of polymethacrylimide (PMI) foam with a thermal conductivity of 0.02 W/(m·K).

The resistive pastes were prepared by incorporating carbon black absorbers into a resin matrix, and two pastes with nominal sheet resistances of 40 Ω/□ and 25 Ω/□ were obtained by adjusting the carbon-black content. A 300-mesh screen was used for screen printing, with the ink knife and squeegee speeds set to 30 mm/s and 25 mm/s, respectively. The resulting resistive-film thickness was approximately 15–20 μm. During fabrication, the lower square-ring and upper cross patterns were fabricated using the 40 Ω/□ and 25 Ω/□ resistive pastes, respectively, followed by curing at 120 °C and 100 °C for 30 min, respectively. The curing process was performed in an oven, and the different curing temperatures were adopted because the two pastes had different formulations and electrical conductivities. After curing, small square test coupons were printed and characterized using a four-point probe to verify the actual sheet resistance of the two resistive films. By separately controlling the sheet resistance and curing conditions of the two layers, the electrical properties of the functional layers were kept close to the design requirements.

Figure 6a shows the fabricated dual-layer periodic resistive-film absorber and the pattern details. The square-ring and cross patterns have clear edges, and the resistive-film layers are uniformly attached, indicating that the designed patterns in both layers are well-realized. The reflection response of the fabricated sample was then measured. During the measurement, the absorber was supported by a wave-transparent honeycomb layer. The edges of the sample were wrapped with adhesive paper tape to reduce the influence of air gaps introduced during assembly. This treatment makes the measurement boundary closer to the simulation model. The measurement system consisted of a vector network analyzer and two broadband double-ridged horn antennas. The reflection response was measured using the free-space arch measurement method over 2–18 GHz, as shown in Figure 6b.

Figure 6c compares the measured and simulated reflection responses. The measured curve shows a similar trend to the simulated one. The main resonance positions also agree well with the design prediction. The measured reflection response remains below −10 dB from 5.1 to 18 GHz, demonstrating good high-frequency broadband absorption. Some differences are still observed between the measured and simulated results, mainly including shifts in the resonance positions and reflection-level deviations in local frequency ranges. The deviations may originate from fabrication tolerances, including sheet-resistance variation, pattern dimensional errors, layer misalignment, and measurement uncertainty. In particular, the upper cross pattern has a relatively low design sheet resistance and is sensitive to parameter variations. Therefore, a small deviation in sheet resistance may lead to a shift of the high-frequency resonance position.

After verifying the broadband absorption performance, low-frequency high-power irradiation tests were further conducted to evaluate the thermal-management capability of the absorber. During the test, the sample was placed in front of a high-power transmitting antenna. Thermocouples were arranged near the irradiated surface to monitor the temperature response under high-power electromagnetic irradiation. The experimental setup is shown in Figure 7a. A representative low-frequency point of 1 GHz was selected for the high-power irradiation test. Because the antenna operates in the near-field region, the irradiation field is not completely uniform, and the local power density on the sample may vary with the antenna aperture, measurement distance, and field distribution.

Figure 7b shows the transient temperature-rise curves of the sample under the 1 GHz irradiation conditions. The antenna output power was adjusted to achieve an estimated incident power density of approximately 20 kW/m^2^. The temperature rises rapidly at the beginning of irradiation and then gradually reaches a stable state. The steady-state temperature at the monitored point reaches approximately 59.7 °C, which demonstrates the thermal-management capability of the absorber under low-frequency high-power irradiation.

Compared with the simulated results, the measured steady-state temperature at 1 GHz is close to the simulated maximum temperature of the lower square-ring layer (61.7 °C). This agreement indicates that the thermal simulation can reasonably capture the low-frequency thermal response. Overall, the experimental results confirm that the designed absorber can effectively control temperature rise under the tested high-power irradiation conditions.

## 6. Conclusions

In this work, a dual-layer periodic resistive-film absorber was developed for broadband microwave absorption operating under high-power antenna irradiation. The absorber was designed to achieve high-frequency broadband absorption while improving low-frequency thermal-management capability. The electromagnetic–thermal responses of cross-shaped and square-ring resistive-film patterns were investigated, revealing that the geometric parameters and sheet resistance regulate heat generation through Ohmic loss and electromagnetic coupling. Guided by these results, a dual-layer absorber consisting of a lower square-ring layer and an upper cross-shaped layer was designed using equivalent-circuit modeling and full-wave simulation. The fabricated absorber achieved a measured reflection below −10 dB from 5.1 to 18 GHz, while the monitored temperature remained at 59.7 °C under 1 GHz high-power irradiation. The proposed strategy provides an effective approach for balancing broadband absorption and thermal-management capability in high-power antenna irradiation environments.

## Figures and Tables

**Figure 1:**
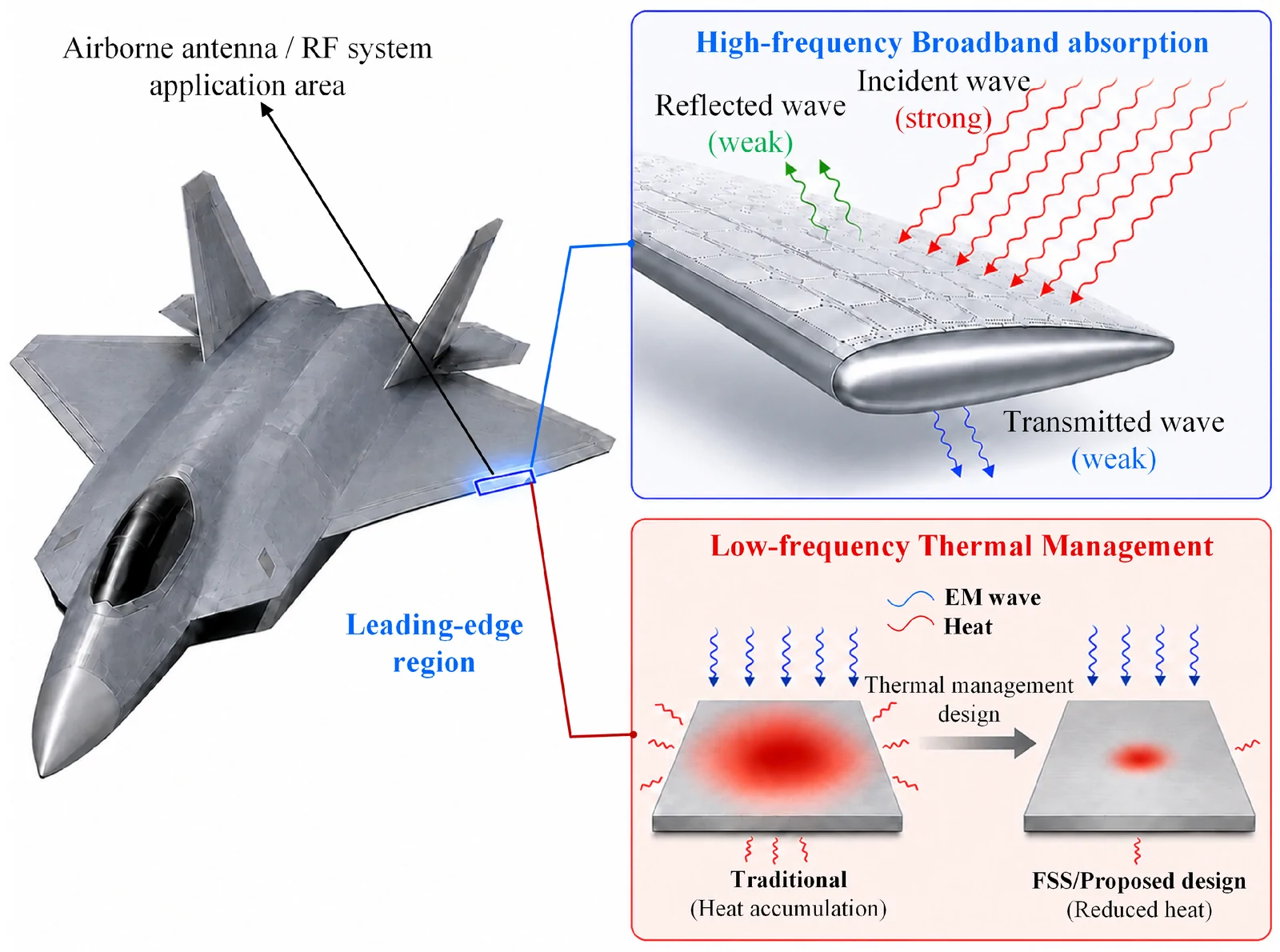
Schematic illustration of broadband microwave absorption and thermal management under high-power antenna irradiation.

**Figure 2:**
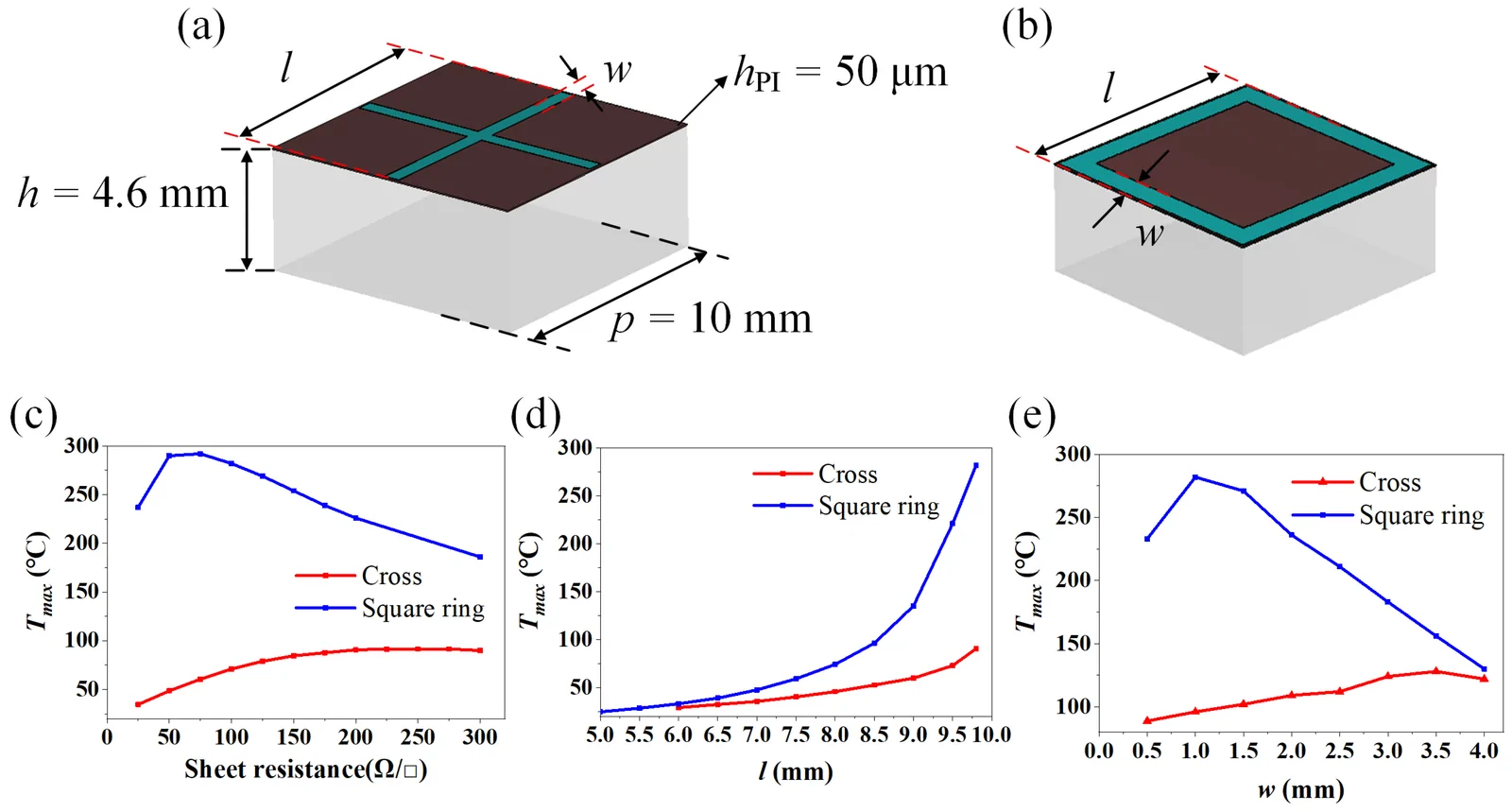
Cross and square-ring periodic resistive-film patterns and thermal responses. The red lines indicate the characteristic dimensions of the patterns. (**a**) Cross pattern. (**b**) Square-ring pattern. (**c**) Maximum temperature varies with sheet resistance. (**d**) Maximum temperature varies with characteristic length *l*. (**e**) Maximum temperature varies with width *w*.

**Figure 3:**
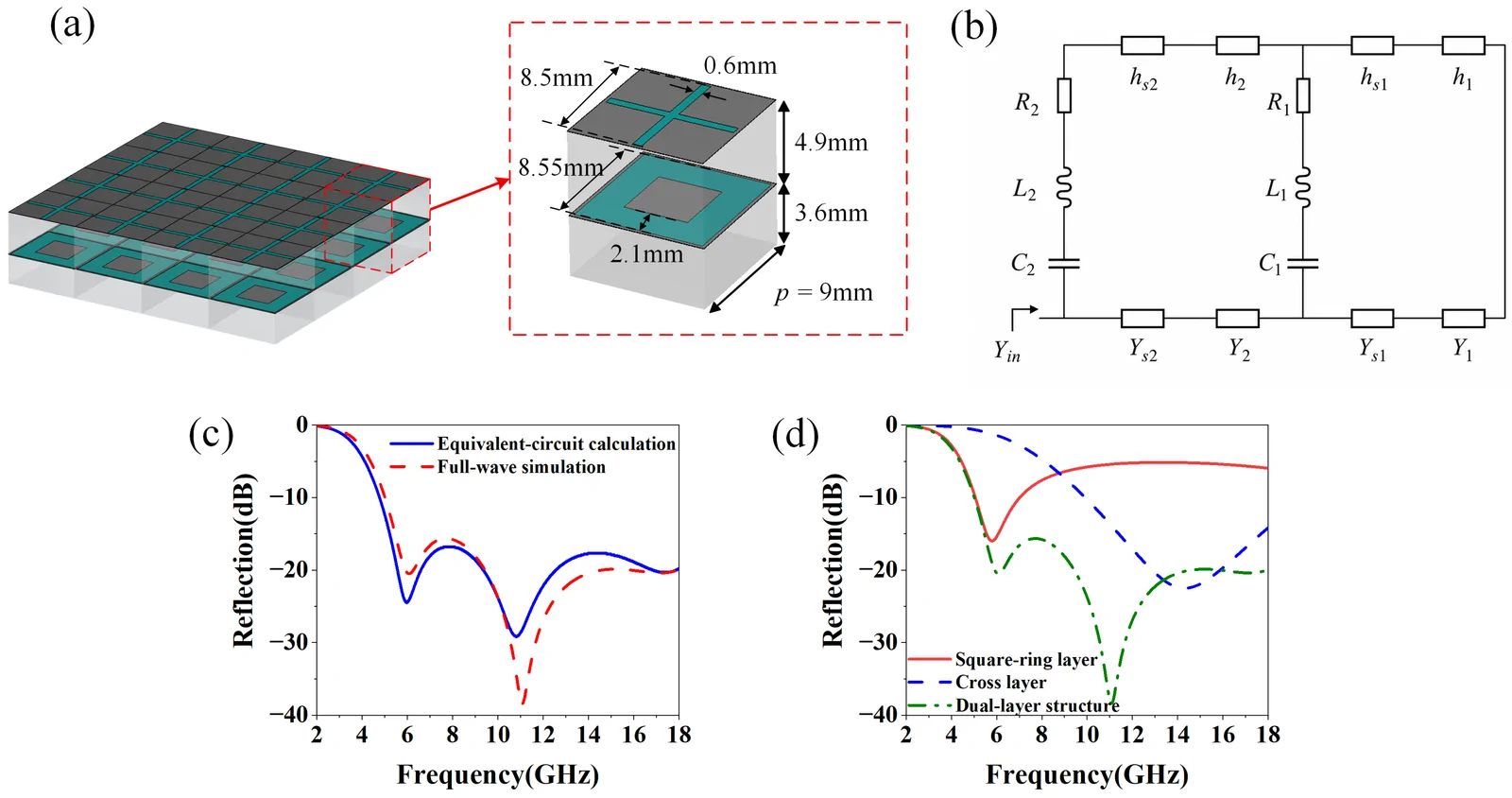
Design and electromagnetic analysis of the dual-layer periodic resistive-film absorber. (**a**) Schematic of the absorber and unit cell. (**b**) Equivalent-circuit model. (**c**) Comparison between equivalent-circuit calculation and full-wave simulation. (**d**) Reflection responses of individual and dual-layer structures.

**Figure 4:**
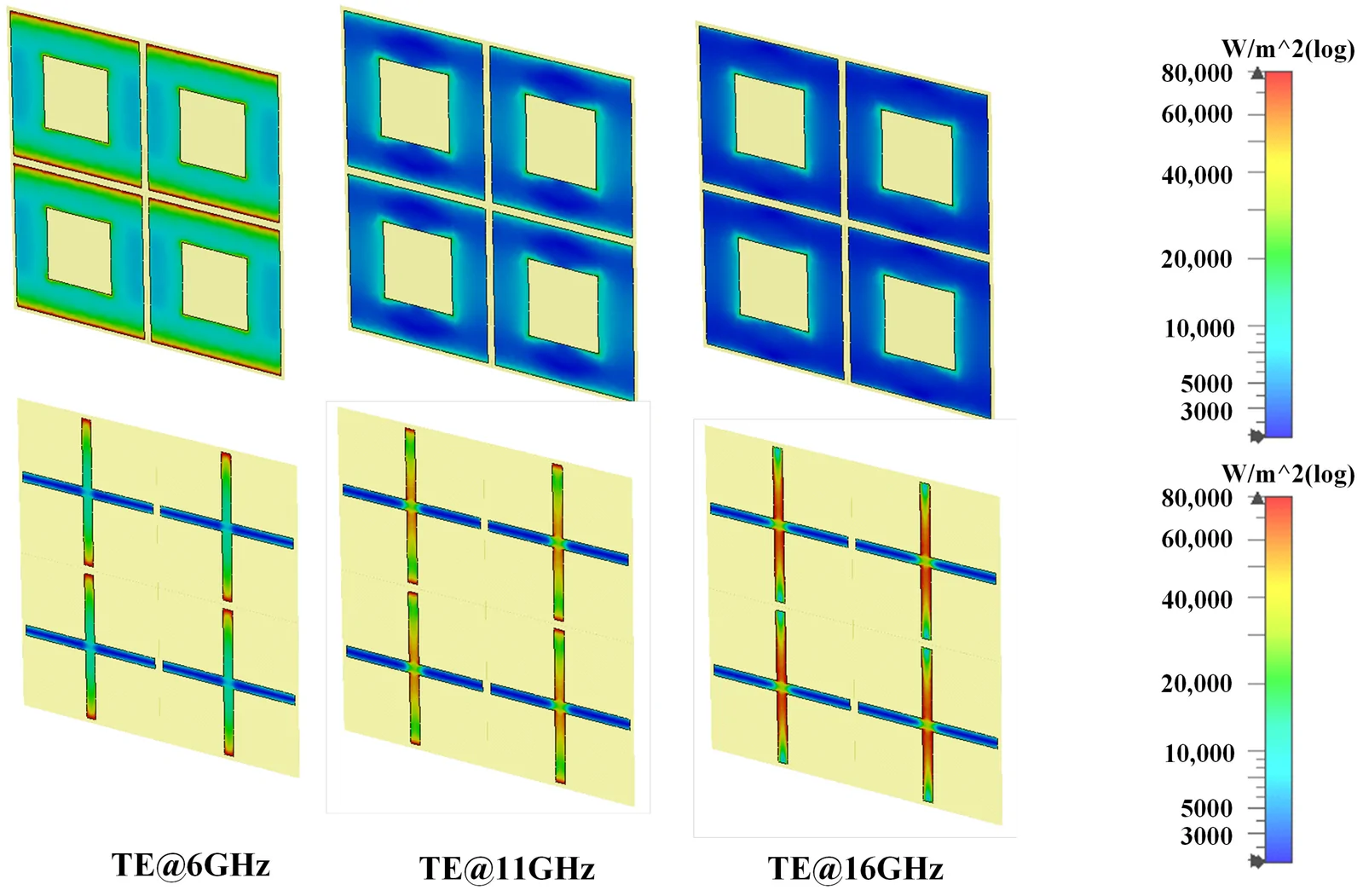
Loss power density distributions in the square-ring and cross layers at 6 GHz, 11 GHz, and 16 GHz.

**Figure 5:**
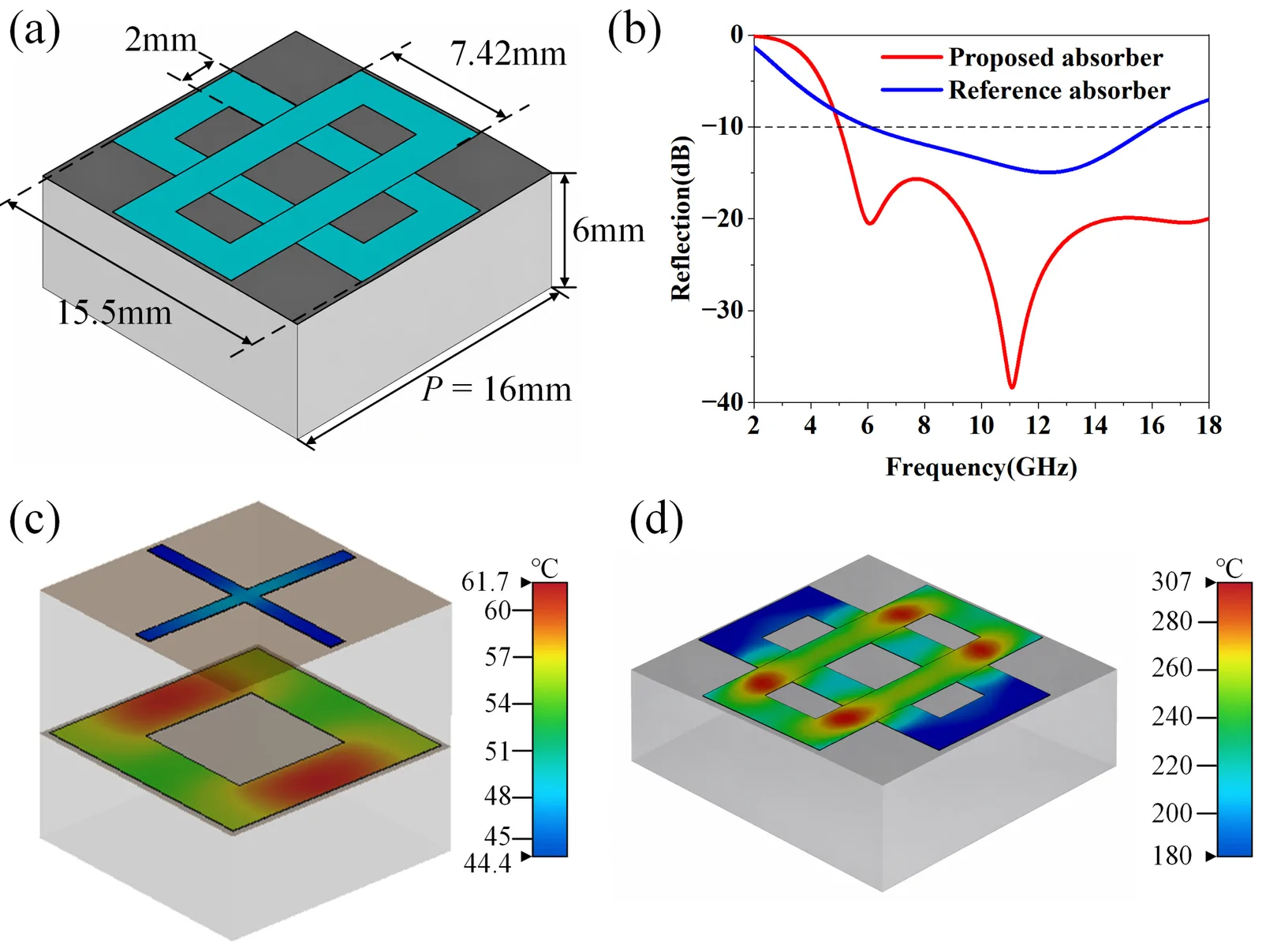
Comparison between the proposed absorber and the reference absorber: (**a**) unit cell structure of reference absorber; (**b**) simulated reflection responses; (**c**) temperature distribution of the proposed absorber; and (**d**) temperature distribution of the reference absorber under 1 GHz irradiation. The blue lines in the structural illustration represent the resistive-film patterns, and the gray dashed line indicates the −10 dB reflection level.

**Figure 6:**
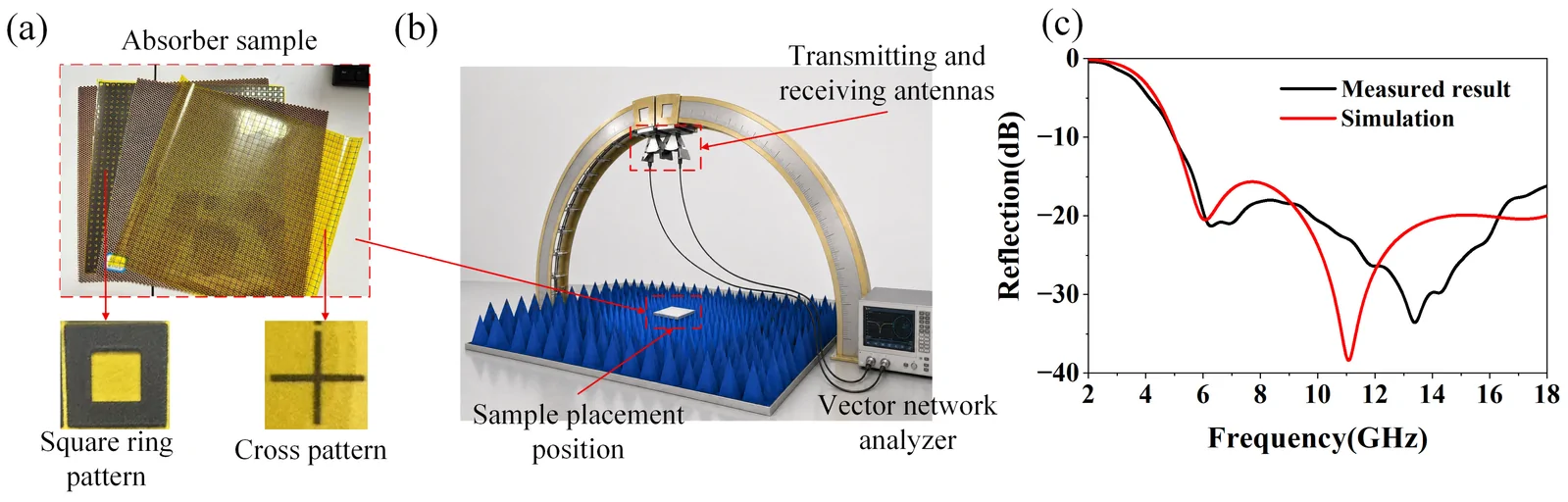
Fabricated absorber and reflection measurement. (**a**) Fabricated sample and pattern details. (**b**) Free-space reflection measurement setup. (**c**) Comparison between measured and simulated reflection responses.

**Figure 7:**
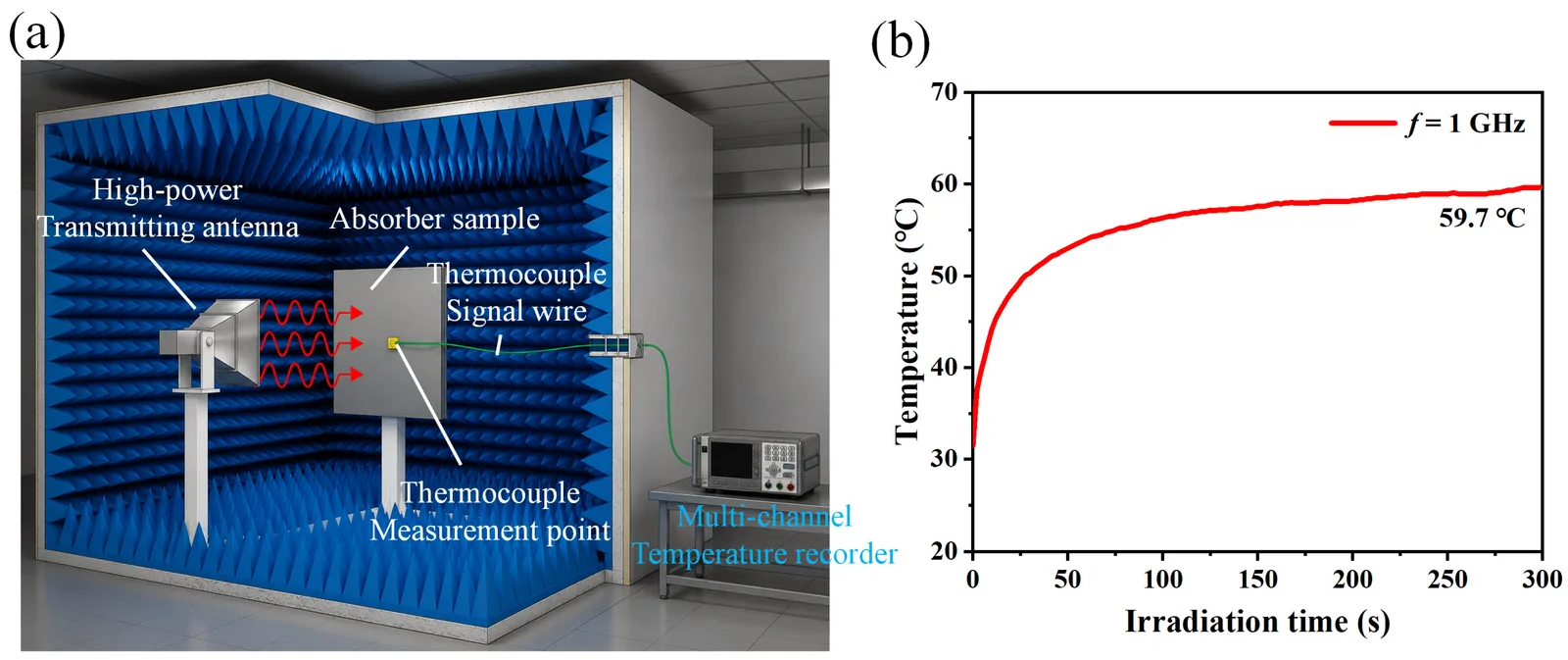
Thermal-response measurement under low-frequency high-power irradiation. (**a**) High-power irradiation test setup. The red arrows indicate the direction of electromagnetic irradiation. (**b**) Transient temperature-rise curves at 1 GHz.

## Data Availability

The original contributions presented in this study are included in the article. Further inquiries can be directed to the corresponding author.

## References

[1] Joy V., Dileep A., Abhilash P.V., Nair R.U., Singh H. (2021). Metasurfaces for Stealth Applications: A Comprehensive Review. J. Electron. Mater..

[2] Che Y.-X., Wu S.-J., Li M., Ban Y.-L. (2025). Research Progress on FSS Stealth Radome. Electronics.

[3] Wang F., Wang Y., Zhang X., Liu L., Li K., Ren Y. (2024). Wideband Low-RCS and Gain-Enhanced Antenna Using Frequency Selective Absorber Based on Patterned Graphene. Sci. Rep..

[4] Fan Q., Huang Y., Chen M., Li Y., Song W., Fang D. (2019). Integrated Design of Component and Configuration for a Flexible and Ultrabroadband Radar Absorbing Composite. Compos. Sci. Technol..

[5] Wang P., Zhang Y., Chen H., Zhou Y., Jin F., Fan H. (2018). Broadband Radar Absorption and Mechanical Behaviors of Bendable Over-Expanded Honeycomb Panels. Compos. Sci. Technol..

[6] Han Y., Liao S., Xiu X., Li B., Chang Y., Xue Q., Che W. (2022). Bandstop Frequency Selective Surfaces Based on Aramid Paper Honeycomb Structure. IEEE Trans. Antennas Propag..

[7] Han Y., Li Q., Li B., Chen J., Xiu X., Che W., Xue Q. (2023). Light-Weight Wideband Electromagnetic Wave Absorber Based on Aramid Paper. Appl. Phys. Lett..

[8] Wang Y., Liang D.-F., Han T.-C., Deng L.-J. (2024). Thermal Management by Manipulating Electromagnetic Parameters. Chin. Phys. B.

[9] Yan H., Fu B., Shan Y., Sun Y., Yao X. (2024). A Fully Coupled Electromagnetic-Thermo-Mechanical Model for Honeycomb Microwave Absorbing Structure. Int. J. Solids Struct..

[10] Li Y., Zhong Y., Wang M., Chen K., Ren P., Xiang Z. (2024). A Miniaturized and Highly Stable Frequency-Selective Rasorber Incorporating an Embedded Transmission Window. Micromachines.

[11] Bu D.-D., Yue C.-S., Zhang G.-Q., Hu Y.-T., Dong S. (2016). Broadband, Polarization-Insensitive, and Wide-Angle Microwave Absorber Based on Resistive Film. Chin. Phys. B.

[12] Weng Z., Li Y., Su Y., Li Z., Guo J., Lv Z., Liang C. (2025). Design and Analysis of Ultra-Thin Broadband Transparent Absorber Based on ITO Film. Micromachines.

[13] Wei J., He Y., Bie S., Wu S., Lei Z., Deng W., Liu Y., Zhang Y., Li C., Ai J. (2019). Flexible Design and Realization of Wideband Microwave Absorber with Double-Layered Resistor Loaded FSS. J. Phys. D Appl. Phys..

[14] Kundu D., Baghel S., Mohan A., Chakrabarty A. (2019). Design and Analysis of Printed Lossy Capacitive Surface-Based Ultrawideband Low-Profile Absorber. IEEE Trans. Antennas Propag..

[15] Li R., Dong J., Si K., He F., Zha D., Miao L., Bie S., Jiang J. (2021). Approaching the Lowest Operating Frequency Thickness Limits with Complex Surface Impedance of Ultrathin Absorbers. Opt. Express OE.

[16] He F., Si K., Li R., Zha D., Dong J., Miao L., Bie S., Jiang J. (2022). Frequency Selective Surface Composites with Honeycomb Absorbing Structure for Broadband Applications. IEEE Trans. Antennas Propag..

[17] Costa F., Monorchio A., Manara G. (2012). Efficient Analysis of Frequency-Selective Surfaces by a Simple Equivalent-Circuit Model. IEEE Antennas Propag. Mag..

[18] Hossain M.I., Nguyen-Trong N., Abbosh A.M. (2022). Broadband Magnetic Absorber Based on Double-Layer Frequency-Selective Surface. IEEE Trans. Antennas Propag..

[19] Choe H.-S., Lee J.-S., Kweon J.-H., Nam Y.-W., Choi W.-H. (2022). High-Performance Microwave Absorption Heating Honeycomb Sandwich Composite with Electroless Nickel-Plated Glass Fiber. Compos. Struct..

[20] Gong P., Liu L., Qi Y., Yu W., Li F., Zhang Y. (2017). Multiphysics Analysis of Millimeter-Wave Absorber with High-Power Handling Capability. IEEE Trans. Electromagn. Compat..

[21] Payne K., Xu K., Choi J.H., Lee J.K. (2021). Multiphysics Analysis of Plasma-Based Tunable Absorber for High-Power Microwave Applications. IEEE Trans. Antennas Propag..

[22] Li M., Behdad N. (2013). Frequency Selective Surfaces for Pulsed High-Power Microwave Applications. IEEE Trans. Antennas Propag..

[23] Lu Y., Chen J., Li J., Xu W. (2022). A Study on the Electromagnetic–Thermal Coupling Effect of Cross-Slot Frequency Selective Surface. Materials.

[24] Bao G., Li P., Sun J., Chen E., Li S. (2024). A High-Temperature-Resistant Stealth Bandpass/Bandstop-Switchable Frequency Selective Metasurface. Micromachines.

[25] Guo M., Sang D., Yuan F., Guo T., Zheng Y., Chen Q., Fu Y. (2022). Broadband Absorptive Frequency-Selective Rasorber Based on Multilayer Resistive Sheets Using Multilayer Resonator. IEEE Trans. Antennas Propag..

[26] Kundu D., Mohan A., Chakrabarty A. (2016). Single-Layer Wideband Microwave Absorber Using Array of Crossed Dipoles. IEEE Antennas Wirel. Propag. Lett..

[27] Qin F., Brosseau C. (2012). A Review and Analysis of Microwave Absorption in Polymer Composites Filled with Carbonaceous Particles. J. Appl. Phys..

